# Supraventricular Tachycardia in Pregnancy: Gestational and Labor Differences in Treatment

**DOI:** 10.7759/cureus.18479

**Published:** 2021-10-04

**Authors:** Crystal N Ibetoh, Eugeniu Stratulat, Fan Liu, George Y Wuni, Ronak Bahuva, Muhammad A Shafiq, Boula S Gattas, Domonick K Gordon

**Affiliations:** 1 Family Medicine, California Institute of Behavioral Neurosciences & Psychology, Fairfield, USA; 2 Medicine, California Institute of Behavioral Neurosciences & Psychology, Fairfield, USA; 3 Internal Medicine, California Institute of Behavioral Neurosciences & Psychology, Fairfield, USA; 4 Internal Medicine, California Institute of Behavioral Neuroscience & Psychology, Fairfield, USA; 5 Internal Medicine, University at Buffalo, Buffalo, USA; 6 Internal Medicine, Rawalpindi Medical University, Islamabad, PAK; 7 Internal Medicine, Scarborough General Hospital, Scarborough, TTO

**Keywords:** supraventricular tachycardia, pregnancy, tachyarrhythmia, maternal, ekg, palpitations

## Abstract

Supraventricular tachycardia (SVT) is a tachyarrhythmia characterized by a heart rate above 120 beats per minute (BPM). Patients with SVT exhibit the following symptoms: palpitations, shortness of breath, chest pain, hemodynamic instability, or possibly asymptomatic. The increase in cardiac output and the increase in resting heart rate during pregnancy predispose pregnant women to SVT. The management of SVT in pregnancy, although remarkably similar, varies slightly based on the trimester of pregnancy. Atenolol and verapamil are effective methods of treating SVT, which can be used during the second and third trimesters. Both medications are contraindicated in the first trimester. At the same time, intravenous adenosine can be used in all three trimesters, including labor. Electrical cardioversion is an effective treatment method for hemodynamically unstable or drug-refractory patients, which has proven to be safe in all three trimesters, including labor but can result in pre-term labor in the third trimester. Non-fluoroscopic ablation proved to be the only treatment method that definitively resolved SVT without recurrence.

## Introduction and background

Supraventricular tachycardia (SVT) is a tachyarrhythmia that involves atrial tissue or atrioventricular junctional tissues [1,2]. This tachyarrhythmia is characterized by a heart rate above 120 beats per minute (BPM) [2]. The conduction pathways' functionality, speed, or blockage determines the type of supraventricular tachycardia [1]. There are approximately four types of SVT; however, atrioventricular reentrant tachycardia (AVRT) and atrioventricular nodal reentrant tachycardia (AVNRT) account for the two most common forms [1,3]. At presentation, the electrocardiogram (EKG) may display narrow QRS complexes with P waves that may be subtle or possibly buried within the ST-segment, inverted P waves, or bizarre QRS complexes [1,2]. 

Additionally, SVT can consist of multiple P wave morphologies, short PR intervals, or undetectable P waves [4]. SVT can be paroxysmal, defined as an abrupt onset and cessation of arrhythmia [4]. Many pregnant patients presenting with this arrhythmia have no previous congenital heart disease or cardiac structural abnormalities [5].

SVT's symptom presentation can consist of palpitations, shortness of breath, hemodynamic instability, or it can be asymptomatic [6]. The etiology of SVT in pregnancy is complicated and multifactorial. The female physiology drastically changes during pregnancy commencing in the first trimester, with an increase in blood volume up to 50% [6-8]. Changes in blood volume contribute to cardiac output that stretches the myocardial tissues predisposing pregnant women to arrhythmias [9,10]. Typically, pregnant women have a considerably increased plasma volume; however, pregnant patients with volume depletion can be predisposed to SVT [7]. Hemodynamic changes in pregnancy result in an increased heart rate by at least 20% during the third trimester due to the fall in systemic vascular resistance [11]. This is displayed by the noticeably higher resting heart rate [12]. High amounts of estrogen have a significant effect on the cardiac tissue's excitability at the molecular level [13]. The vast increase in catecholamines' circulating levels results in additional adrenergic receptors leading to an arrhythmia [12-14]. 

The effective management of SVT in pregnancy depends on the presentation of symptoms and the trimester of pregnancy. The patient may present with hemodynamic instability resulting in a more aggressive treatment approach [15]. Milder cases may go undetected or may be treated with vagal maneuvers [16]. When vagal maneuvers fail, medical management becomes the preferred treatment of choice [17]. SVT can present at any stage of pregnancy, including labor [18]. Due to this fact, the safest form of treatment should be considered to ensure the mother's safety and prevent adverse effects on the fetus [19]. Medications or treatments used during the second or third trimester may be contraindicated in the first trimester [19]. Unique treatments and diagnostic tools have been used to treat pregnant patients with SVT. One such treatment modality, which will be examined in this article, is catheter ablation without fluoroscopy [20]. Researchers have found multiple ways to achieve catheter ablation and reduce fetal radiation exposure. This literature review article aims to examine each treatment modality during each trimester of pregnancy, including labor, and determine if additional research should be conducted to improve these methods.

## Review

Discussion

First Trimester 

The first trimester consists of the first 13 weeks of pregnancy. During the first trimester of pregnancy, numerous physiological changes occur within the body. As mentioned previously, the blood volume nearly doubles increasing cardiac output [9]. The fetus's safety should be heavily considered during this trimester as organogenesis takes place within the fifth to tenth week of gestation [1,9]. The fetus is extremely vulnerable to congenital disabilities, and the mother is highly susceptible to spontaneous abortion during this period [21]. In cases in which the patient is hemodynamically unstable, the immediate benefit of the treatment outweighs the possible teratogenic effects.

Based on a thorough examination of the literature, vagal maneuvers are considered the best initial treatment for SVT [9]. This non-pharmacological treatment is well-tolerated in the first trimester. Vagal maneuvers consist of carotid sinus massage, Valsalva, and facial ice immersion. This form of treatment should be used in hemodynamically stable patients. Although vagal maneuvers are considered extremely safe during pregnancy, it may not be the most effective treatment. In a study conducted by Jian-Ming Li et al., SVT was terminated with Valsalva maneuver in 3% of patients [22]. If vagal maneuvers do not resolve the SVT, pharmacological treatments must be considered. 

Adenosine is the first-line pharmacological agent to treat SVT. Intravenous (IV) adenosine is unlikely to enter fetal circulation because it has a tremendously short half-life of 10 seconds. This medication is typically administered intravenously with 6 mg to 12 mg. This medication has been successful in 84% of cases [23]. Beta-adrenergic blockers, such as propranolol and metoprolol, are anti-arrhythmia drugs that can be first-line for outpatient treatment and second-line for acute treatment [12]. Digoxin has been used in the first trimester of pregnancy and is relatively safe [23]. If the patient's arrhythmia is drug-refractory or if the patient is hemodynamically unstable, electric cardioversion can be used to terminate SVT. Electric cardioversion has been proven to be safe in all stages in life-threatening circumstances. Synchronized cardioversion is typically administered at 50 to 400 J [24]. 

Many antiarrhythmic medications are incredibly useful in non-pregnant patients, but a few pose a significant risk to the fetus. One such cardioselective beta-1-adrenergic blocker that must be avoided in the first trimester of pregnancy is atenolol [25]. It is a class D drug and results in intrauterine growth retardation. In addition to atenolol, amiodarone should only be reserved for life-threatening circumstances. It is considered a class D medication and is typically used in conjunction with electric cardioversion [26]. Verapamil is a non-dihydropyridines calcium channel blocker that should be avoided in the first trimester of pregnancy because it can enter the fetal circulation and cause fetal arrhythmias such as fetal bradycardia and heart block [27].

Catheter ablation is the most effective treatment of SVT, even in the first trimester of pregnancy. This modality is generally reserved for treatment refractory cases or when pharmacological treatments are contraindicated or refused [20]. The traditional form of catheter ablations involves the radiological modality called fluoroscopy. Fluoroscopy is utilized to position the catheter in the heart [28]. This presents a significant problem as the X-ray emissions from this procedure are dangerous to the fetus [29]. Non-fluoroscopic ablation must be used in order to prevent any harm to the fetus. Non-fluoroscopic ablation can be achieved using three-dimensional electroanatomical mapping or intracardiac echocardiography [26,30]. 

Three-dimensional electroanatomical mapping uses CARTO 3 Version 4 software (Biosense Webster Inc., CA, USA) to locate the area of the heart that will be ablated. A decapolar catheter with steerable capabilities is placed in the appropriate location within the heart. In a case study conducted by Dr. Georgy Kaspar et al., decapolar catheters were inserted into the coronary sinus and the crista terminalis of an 11-weeks pregnant female [26]. The origin of the arrhythmia was believed to be located at the crista terminalis or superior vena cava. A Schwartz Right 0 sheath, which is used to stabilize the catheter, achieved successful ablation, with a contact force of 10 to 20 g and ablation at 30 Watts [26,31]. Non-fluoroscopic ablations can be achieved with intracardiac echocardiography to locate the structures in Koch's triangle. Bongiorni et al. completed a radiofrequency ablation with intracardiac echocardiography in 10-week pregnant women [30]. The intracardiac echocardiography (ICE) was attained using a 9F-MHz Ultra ICE catheter-based transducer [30]. This ablation procedure was completed using a 55˚ pre-curved polyethylene long venous sheath, which guided the ICE catheter, a quadripolar catheter, and 4-mm-tip ablation catheter. The ablation took place at the posterior of Koch's triangle, and it successfully terminated the arrhythmia [30].

Many studies discouraged the use of any pharmacological treatment during the first trimester of pregnancy. It is believed that the use of pharmacological medications during the first eight weeks of pregnancy could negatively affect the embryo when organogenesis is taking place [32]. Although no complications were observed in the two non-fluoroscopic catheter ablation cases listed above, the pregnancies' outcomes were not documented [26,30].

Second Trimester

According to the American College of Obstetricians and Gynecologists (ACOG), the second trimester occurs between the 14th and 27th week of gestation [33]. During this stage of pregnancy, the fetal heart rate can be auscultated, and fetal malformations can be detected through ultrasound, and fetal movement is detected by the mother [34]. Lack of fetal movement can be the mother's first indication of a fetus's problem, unlike the first trimester. There are fewer limitations in treatment during the second trimester because organogenesis is completed by this pregnancy stage [8]. The risk of spontaneous abortion and fetal loss is substantially lower than in the first trimester [21]. Certain medications that are contraindicated in the first trimester can be used chronically or acutely during this trimester. We will examine all the treatment options.

The treatment of SVT during the second trimester of pregnancy is remarkably akin to the first and third trimester. The first-line treatment of SVT in pregnancy is vagal maneuvers, which were described previously. SVT refractory to maneuvers can be treated with IV adenosine that has a high likelihood of terminating SVT [23]. This medication is only indicated for acute treatment and, as mentioned above, has an extremely short half-life. Over a 60-year span of time, IV adenosine administration has resulted in 8% of maternal adverse effects and 6% of fetal adverse effects [23]. The maximum amount of IV adenosine that can be administered is 24 mg [35]. SVT refractory to IV adenosine can be treated with beta-adrenergic blockers. Beta-adrenergic blockers can be utilized not only acutely but also in a chronic setting. In some cases, beta-adrenergic blockers are used solely for chronic treatment. Agrawal et al. initially terminated the SVT of a 26-week pregnancy patient with IV adenosine and discharged the patient on chronic treatment with labetalol at the lowest therapeutic dosage [14]. 

**Figure 1:**
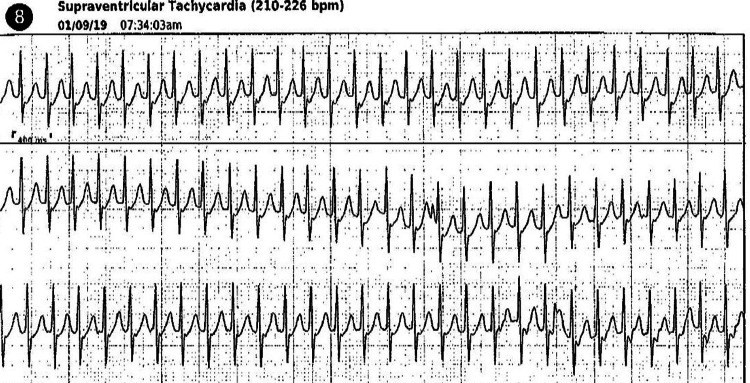
SVT in 38-year-old at 27 weeks of gestation Mobile cardiac telemetry monitor detected SVT at 210 to 226 BPM (medical information was retrieved with the permission of the patient). BPM = Beats per minute, SVT = Supraventricular tachycardia

Intravenous adenosine is indicated as a first-line pharmacological treatment, but it is not always used first-line. A 38-year-old at 27 weeks of gestation presented to the emergency room with a heart rate between 160s-170s, with dizziness and dyspnea. Prior to her emergency room admission, her mobile cardiac device recorded 210-226 BPM (Figure 1). Initially, she was treated with IV metoprolol 5 mg that had minimal response. This medication was repeated twice more with a similar effect. The patient's SVT was officially terminated using IV diltiazem 4 mg. Non-dihydropyridines calcium channel blockers such as diltiazem and verapamil are often avoided during the first trimester but can be used in the second trimester. This medication is not the first or second-line because it can result in maternal hypotension and fetal bradycardia [8]. Maternal hypotension is generally associated with rapid IV administration [36]. Calcium channel blockers, specifically verapamil, can be used as a prophylactic treatment in patients with symptoms that persist [8]. 

The above medications are more effective at treating SVT in pregnancy with a better safety profile than the following medications. Digoxin can be used during the second trimester, but it may not be useful as a single agent [8]. Flecainide is a class IC antiarrhythmic drug that has been used in the second trimester [23]. There are minimal studies that have been conducted with this medication. In one study of a 27-year old patient at 22 weeks of gestation, flecainide failed to terminate the arrhythmia [20]. After reviewing the literature, it has been determined that amiodarone should be reserved as a last resort drug in each trimester [8].

Electrical cardioversion is safe to use in the second trimester. It continues to be reserved for patients who fail to respond to medications or become hemodynamically unstable [32]. Electrical cardioversion is generally successful between 50 J to 100 J. A 32-year-old woman at 26 weeks of gestation presented to the emergency room with chest pain and palpitations [24]. She had a heart rate of 230 BPM. The EKG determined she in fact, had SVT. The patient was treated with vagal maneuvers, IV metoprolol, and IV adenosine without resolve [24]. After the patient became hypotensive and neurologically compromised, it was determined that synchronized cardioversion was the best course of action [24]. Ultimately, the cardioversion terminated the SVT.

Catheter ablations are generally indicated for treatment-refractory SVT. This procedure is safe to administer during the second trimester of pregnancy. A non-fluoroscopic ablation was achieved on a 22-year-old woman at 25 weeks of gestation [37]. The catheter ablation procedure was navigated by the Ensite NavX system as opposed to fluoroscopy [37]. Mapping catheters are placed at the proper anatomical location to determine the precise mapping points. In this patient, the precise mapping points were located within the tricuspid valve [37]. The accessory pathway causing the arrhythmia was successfully ablated after three radiofrequency applications were delivered to the precise mapping points within the tricuspid valve [37]. Non-fluoroscopic ablation completed with 3D-dimensional mapping has been proven successful in the second trimester on a 22-year-old woman at 22 weeks of twin gestation. Both patients had favorable outcomes and no recurrence of SVT [20]. 

Third Trimester

According to ACOG, the third trimester of pregnancy occurs between the 28th and 40th week of pregnancy [33]. During the final trimester of pregnancy, the fetus's respiratory system becomes fully developed, and the fetus has increased viability [34]. In a study conducted by Li et al., it was determined that a large percentage of patients developed SVT during the third trimester [38]. An arrhythmia's likelihood remains high due to major hemodynamic changes that impact the body [39]. Additionally, the increased heart during the third trimester predisposes patients to any arrhythmia [8]. Unlike the second trimester of pregnancy, the fetal loss is less likely to occur during this trimester. Pre-term infants have a higher chance of survival outside of the womb during this trimester. Some of the medical interventions beneficial in the first and second trimester result in pre-term labor during the third trimester.

Vagal maneuvers remain the first-line non-pharmacological treatment of SVT in all trimesters. After a thorough literature review, it has been determined that vagal maneuvers have proven to be well-tolerated in all trimesters of pregnancy [13]. A 33-year-old at 37 weeks of gestation presented to the emergency room with a heart rate of 240 BPM [13]. She was initially treated with carotid sinus massage, which reduced her heart rate but did not terminate the arrhythmia. After a second IV bolus of IV adenosine, her SVT was terminated [13]. Adenosine is the first-line pharmacological treatment in all trimesters of pregnancy. It has a higher conversion rate than beta-adrenergic and calcium channel blockers [23]. Despite its effectiveness, adenosine has been linked to pre-term labor during the third trimester [5]. A patient at 30-weeks of gestation was successfully treated with IV adenosine. However, immediately after the administration of IV adenosine, uterine contractions commenced [40]. Fortunately, tocolysis was achieved with the use of calcium channel blockers [40]. 

Although IV adenosine has a higher conversion rate than beta-adrenergic blockers and calcium channel blockers, these drugs do not result in pre-term labor. They are effective and used in the third trimester. Propranolol, metoprolol, and sotalol have all been used to treat SVT acutely and chronically [27,39]. In the case of a 38-year-old female who presented with SVT at 27 weeks of gestation, she was chronically managed throughout her third trimester on low-dose metoprolol. This patient did not suffer any adverse effects from chronic management. While beta-adrenergic blockers are an effective treatment, it was observed that in the majority of the third trimester's cases we reviewed, the patients did not revert to normal sinus treatment with this pharmacological treatment. The one advantage calcium channel blockers have over adenosine is that they do not cause uterine contraction [40]. Calcium channel blockers happen to be tocolytic agents. 

Disopyramide is a class IA antiarrhythmic agent. This medication has a lower conversion rate and causes uterine contractions and hemorrhage [23]. For this reason, this medication is generally not used as a first or second-line agent. A 26-year-old woman in her third trimester with persistent SVT was treated with disopyramide. As a result, the patient developed uterine contractions and hemorrhage [39]. Based on the literature review, the use of disopyramide is not recommended because of the previously mentioned adverse effects [23].

When the patient is hemodynamically unstable or unresponsive to pharmacologic agents in acute circumstances, electrical cardioversion has been used during the third trimester. Electrical cardioversion is safe and overall has been well-tolerated in all three trimesters. Electrical cardioversion successfully treated a 30-year-old at 37 weeks of gestation with drug-refractory SVT and hemodynamic instability [14]. A small risk of pre-term labor is connected to electrical cardioversion in the third trimester [8]. Electrical cardioversion has stimulated labor and resulted in fetal distress. A 24-year-old woman at 28 weeks of gestation, after multiple treatments were attempted, developed hypotension, chest pain, and heart rate increased to 200 BPM [39]. Synchronized cardioversion at 50 J was delivered, which terminated the SVT. Following this action immediately, uterine contractions and fetal bradycardia occurred, resulting in an emergent cesarean section [39]. 

A comprehensive literature review has determined that catheter ablation in all three trimesters has proven to be the most effective treatment for this arrhythmia. It has been proven to be safe with long-lasting results. There are three cases in which third-trimester patients were treated with radiofrequency ablation with the use of fluoroscopy. Lead abdominal protection was used to shield the uterus from radiation exposure. In total, they were exposed to approximately 6.8 to 29.6 minutes [29]. Two out of the three patients gave birth to healthy babies without fetal abnormalities or congenital illnesses. One patient's child was diagnosed with microencephaly, which was not linked to radiation exposure [29]. These patients were exposed to minute amounts of radiation. Despite normal fetal outcomes, the possibility of an increased risk of leukemia should not be ruled out in each case. Radiation exposure is oncogenic to the unborn fetus [29]. 

Low-dose or non-fluoroscopic ablations are the preferred methods of catheter ablation. We will examine the case of a patient in their third trimester who successfully underwent a non-fluoroscopic ablation. An 18-year-old woman at 33 weeks of gestation was treated with catheter ablation after she failed to respond to IV metoprolol, diltiazem, and digoxin and became significantly unstable [41]. Catheter ablation was performed with minimal fluoroscopy using an intracardiac endocardiography catheter and a CARTO 3 (Biosense Webster Inc., CA, USA) electroanatomic mapping system [41]. Approximately 1.3 minutes of fluoroscopy were used solely to confirm the positioning of the transeptal needle [41]. No adverse effects occurred during or after the procedure to the mother or child.

Labor & Delivery

SVT can present during labor. Romem et al. conducted a study that determined cardiac arrhythmias occur more often during labor [42]. The cause of SVT during labor is multifactorial. Common triggers of SVT during labor are the catecholamine release, electrolyte disturbances such as hyperkalemia, and vasopressors administered to treat post-epidural hypotension [13]. The cardiac output of a pregnant woman at term increases to approximately 10 liters at term [16]. As previously stated, the increased cardiac output results in stretches on myocardial tissue developing into this tachyarrhythmia [9,10]. SVT can present at any time during the labor process. How is SVT managed when it presents during labor? We will examine a few cases of SVT during labor.

A 40-year-old woman at term was admitted to the hospital for induction of labor [10]. Three methods were used to induce labor, including artificial rupture of the membranes, topical prostaglandins, and oxytocin. After hours passed by, the patient developed palpitations [10]. It was later determined after an EKG was completed that she had atrioventricular nodal reentrant tachycardia (AVNRT), a form of SVT. The initial treatment with vagal maneuvers failed to terminate the SVT. IV adenosine ultimately terminated the SVT [10]. The patient delivered a healthy baby via caesarian section with no reoccurrence of SVT during the post-partum period. 

A 31-year-old woman at 38 weeks of gestation was admitted to the hospital with spontaneous labor. An EKG was performed after the patient complained of dizziness and an "unwell" feeling. 13] SVT at a heart rate of 160 BPM was determined at this time [13]. No pharmacological treatments or vagal maneuvers were used to combat the SVT. The physicians believed the best approach was to deliver the patient via caesarian section [13]. Sinus rhythm was achieved after the surgical procedure. A 33-year-old woman at eight days past her due date, presented to the hospital in labor [18]. The patient's initial heart rate was 225 BPM and eventually decreased to 105 BPM. Nine hours after admission, the patient's heart remained at 200 BPM, and SVT was revealed through EKG. The physicians administered an epidural hoping to resolve the SVT [18]. Unfortunately, this method proved to be ineffective. The patient was treated with IV adenosine, IV metoprolol (administered twice), and finally IV verapamil [18]. The IV verapamil ultimately resulted in the termination of the SVT. The patient remained utterly asymptomatic and hemodynamically stable despite having the tachyarrhythmia.

There is limited research on SVT during labor. The management of SVT resembles the management of SVT during the second and third trimesters. Some obstetricians believe that caesarian delivery is a safe approach to SVT patients due to higher catecholamine release that occurs with vaginal deliveries [18]. As mentioned above, the catecholamine release is a trigger of SVT. Anesthesiologists think that epidural anesthesia can maintain minor control of the heart rate by controlling pain [18]. Administration of epidural anesthesia to control the heart can be counterproductive. Spinal anesthesia can result in severe hypotension, leading to the administration of vasopressors to increase the blood pressure to a normal range. Vasopressors such as ephedrine can, once again, prompt the onset of SVT [18]. Although the research on SVT during labor is limited, Table 1 details all the treatment methods that have been used during labor and each trimester of pregnancy. 

**Table 1:** Treatments of SVT based on trimester This table lists treatments used for acute or chronic management of SVT by the trimester and their possible effects on the body. 1^st^ = First, 2^nd^ = Second, 3^rd^ = Third, SV = Supraventricular tachycardia

Treatment method	Trimester of pregnancy	Side/Adverse effects	Result of treatment
Digoxin	1^st^ & 2^nd^	Low birth weight	Low conversion as a single agent
Vagal maneuvers	1^st^, 2^nd^ & 3^rd^	Well tolerated	Low conversion rate
Electrical cardioversion	1^st,^ 2^nd^ & 3^rd^	Preterm labor and fetal distress	High conversion rate
Non-fluoroscopic catheter ablation	1^st^, 2^nd^ & 3^rd^	Well tolerated	High conversion rate
Propranolol	1^st^, 2^nd^ & 3^rd^	Bradycardia^[9]^, uterine growth retardation^[9]^	Effective for outpatient management
Amiodarone	1^st^, 2^nd^ & 3^rd^	Uterine growth retardation	Higher conversion rate with electrical cardioversion
Adenosine	1^st^, 2^nd^, 3^rd^ & labor	Pre-term labor	High conversion
Metoprolol	1^st^, 2^nd^, 3^rd^ & labor	Bradycardia^[9]^, uterine growth retardation^[9]^	Moderate conversion rate. Also effective for outpatient management
Flecainide	2^nd^	Well-tolerated	Low conversion rate
Verapamil	1^st^, 2^nd^, 3^rd^ & labor	Fetal bradycardia^{9] ^and heart block^[9]^, maternal hypotension	High conversion rate. Also effective for outpatient management
Diltiazem	1^st^, 2^nd^, 3^rd^ & labor	Maternal hypotension	High conversion rate
Disopyramide	3^rd^	Uterine contractions and hemorrhage	Low conversion rate

## Conclusions

During the first trimester, second trimester, third trimester, and labor, SVT's management is simple. The management is heavily based on the patient's symptoms, the stage of pregnancy, and the response the body has to the treatment. After extensively reviewing the literature, non-fluoroscopic catheter ablation has proven to be a definitive treatment of SVT in pregnant and non-pregnant patients. It is the one treatment that is safe to use in each trimester without any adverse effects. 

Should a caesarian section be chosen over vaginal delivery to prevent SVT during labor? Additional studies are required to determine the best method to treat SVT during labor since information on this topic is limited. There were numerous challenges in locating articles on this topic. Overall, there were limited large-scale studies conducted on SVT in pregnancy. Most of the literature consisted of case reports with only a few patients. In the future, retrospective and randomized clinical trials without a placebo should be conducted to determine the safest and effective SVT treatment method in all stages of pregnancy.
